# PIG-A gene mutation as a genotoxicity biomaker in polycyclic aromatic hydrocarbon-exposed barbecue workers

**DOI:** 10.1186/s41021-021-00230-1

**Published:** 2021-12-09

**Authors:** Yiyi Cao, Jing Xi, Chuanxi Tang, Ziying Yang, Weiying Liu, Xinyue You, Nannan Feng, Xin Yu Zhang, Jingui Wu, Yingxin Yu, Yang Luan

**Affiliations:** 1 School of Public Health, Shanghai Jiaotong University School of Medicine, Shanghai, 200025 People’s Republic of China; 2 Center for Disease Control and Prevention of the Changning District of Shanghai, Shanghai, 200051 People’s Republic of China; 3 Guangdong-Hong Kong-Macao Joint Laboratory for Contaminants Exposure and Health, Guangdong Key Laboratory of Environmental Catalysis and Health Risk Control, Institute of Environmental Health and Pollution Control, Guangdong University of Technology, Guangzhou, 510006 People’s Republic of China; 4 Guangzhou Key Laboratory of Environmental Catalysis and Pollution Control, Key Laboratory of City Cluster Environmental Safety and Green Development, School of Environmental Science and Engineering, Guangdong University of Technology, Guangzhou, 510006 People’s Republic of China

**Keywords:** Polycyclic aromatic hydrocarbons, *PIG-A* assay, Lymphocyte cytokinesis-block micronucleus test, Genotoxicity

## Abstract

****Background**:**

The *PIG-A* gene mutation assay is a valuable tool for measuring in vivo gene mutations in blood cells. The human *PIG-A* assay, used as a potential genotoxicity biomarker, is minimally invasive, sensitive, and cost-efficient; however, the relationship between carcinogen exposure and *PIG-A* mutations is not well understood.

****Methods**:**

We investigated the genotoxic effect of red blood cells using *PIG-A* assay and lymphocyte cytokinesis-block micronucleus test in barbecue restaurant workers (*N* = 70) exposed to polycyclic aromatic hydrocarbons (PAHs) and self-identified healthy control subjects (*N* = 56). Urinary PAH metabolites were measured to evaluate internal exposure levels.

****Results**:**

Multivariate Poisson regression showed that the PAH-exposed workers exhibited significantly higher *PIG-A* mutant frequency (MF) (8.04 ± 6.81 × 10^− 6^) than did the controls (5.56 ± 5.26 × 10^− 6^) (RR = 0.707, 95% CI: 0.615–0.812, *P* < 0.001). These results indicate that PAH exposure is a risk factor for elevated *PIG-A* MF. The frequencies of micronuclei (MN) and nuclear buds (NBUD) in the PAH-exposed workers (MN: 3.06 ± 2.07 ‰, NBUD: 1.38 ± 1.02 ‰) were also significantly higher than in the controls (MN: 1.46 ± 0.64 ‰, *P* < 0.001; NBUD: 0.70 ± 0.60 ‰, *P* < 0.001). Additionally, *PIG-A* MFs showed better associations with several urinary hydroxylated PAH metabolites (*P*_2-OH-Flu_ = 0.032, r_2-OH-Flu_ = 0. 268; *P*_2-OH-Phe_ = 0.022, r_2-OH-Phe_ = 0.286; *P*_3-OH-Phe_ = 0.0312, r_3-OH-Phe_ = 0.270; *P*_4-OH-Phe_ = 0.018, r_4-OH-Phe_ = 0.296), while the increase in MN, NPB, and NBUD frequencies was not associated with any OH-PAH metabolites; and high-PAH-exposed workers showed the highest *PIG-A* MFs. Furthermore, there was a significant association between *PIG-A* MF and PAH exposure levels (Chi-square test for trend, *P* = 0.006).

**Conclusions:**

Our results indicate that an increase in *PIG-A* MF in barbecue workers could reflect the response to PAH exposure, providing evidence of its potential as a genotoxicity biomarker in human risk assessment.

**Supplementary Information:**

The online version contains supplementary material available at 10.1186/s41021-021-00230-1.

## Introduction

Mutations induced in somatic and germ cells promote human diseases other than cancer; senescence is attributed to the accumulation of deleterious mutations. Mutations induced by exogenous substances, such as genotoxic exposure-induced genetic damage, affect human health. Thus, using mutations as toxicological endpoints to evaluate genetic damage is useful for human health risk assessment. In the last 10 years, a high-throughput flow cytometry-based rodent phosphatidylinositol glycan class A (*Pig-a*/*PIG-A*) mutation assay has been well established. This X-linked gene encodes the catalytic subunit (N-acetylglucosaminyltransferase A), involved in an early step of the biosynthetic pathway for glycosylphosphatidylinositol (GPI) anchors [1–3]. The *Pig-a* mutation assay is not only minimally invasive and cost-effective but is also remarkably sensitive to genotoxic agents [4]. The *Pig-a*/*PIG-A* gene is considered highly conserved among species as a potential sentinel gene and can reflect the somatic mutation rate [1, 5]. Recent studies have indicated the potential of extending the rodent *Pig-a* assay to the human erythrocyte *PIG-A* assay [6–9]. A few studies have reported the spontaneous background *PIG-A* mutant frequency (MF) in self-identified healthy subjects; we have also evaluated its associations with several factors (sex, age, smoking status) [8].

Several studies have recently investigated the effect of *PIG-A* MF induction on genotoxic chemotherapy or radiation regimens in cancer patients to determine whether *PIG-A* MF could be used as a novel biomarker for monitoring genotoxicity in humans. However, these relationships are not well understood, possibly due to the complex nature of the disease and combination therapy [10–13]. We then selected populations occupationally exposed to genotoxicants for further evaluation. Our previous study on occupational lead-exposed workers showed that *PIG-A* MFs were related to a cumulative blood lead index [14]. However, lead is not a potent mutagen, and non-genotoxic modes are also involved in carcinogenesis [15, 16]. In the present study, we investigated the risk associated with the occupational exposure of polycyclic aromatic hydrocarbons (PAHs), a diverse class of organic compounds consisting of two or more fused aromatic rings. Several PAHs, such as benzo [a] pyrene, Benz [a] anthracene, and Dibenz [a,h] anthracene are well-studied carcinogens. For instance, PAHs target bone marrow and promote DNA adduct formation and gene mutations [17, 18]. Occupational exposure to PAHs can increase the risk of various cancers [19, 20]. Epidemiology studies have revealed a significantly higher micronuclei (MN) frequency in the PAH-exposed population [21, 22]. Meanwhile, benzo [a] pyrene has been shown to produce a positive response in the rodent *PIG-A* assay [23–26] Overall, PAHs are mutagenic, and occupational exposure may enhance *PIG-A* MF in humans.

In the present study, we recruited barbecue (BBQ) restaurant workers and healthy control subjects. The fumes generated from BBQ activities contain high PAH levels [27], and inhalation is the most critical pathway of PAH exposure during cooking [28, 29]. We measured urinary PAH metabolites to evaluate the internal exposure level of PAHs and investigated the genotoxic effects on human red blood cells (RBC) using a *PIG-A* assay and a human lymphocyte cytokinesis-block micronucleus (CBMN) test, demonstrating the relationships between the levels of PAH exposure and *PIG-A* MF/MN frequency.

## Materials and methods

### Study population and sample collection

We recruited a total of 126 self-identified healthy subjects including 70 BBQ restaurant workers as PAH-exposed group and 56 hotel administrative staffs without work-related exposure to PAHs as unexposed controls in 2018–2020 in Shanghai, China. According to the different types of work in the BBQ restaurants with different PAHs exposure level, the chefs were considered as the high PAH-exposed group (*N* = 24), the workers including waiter, cashier, manager and others were considered as the low PAH-exposed group (*N* = 46). Ethical approval was granted by the Ethics Committees of Shanghai Jiao Tong University School of Medicine and Center for Disease Control and Prevention of the Changning District of Shanghai (No. CNKW2017Y22). The PAHs exposure of these BBQ restaurant workers primarily occurred through inhalation of airborne particles. There are two types of BBQ restaurant in this study. Type 1: the BBQ food was mainly cooked by chef in the kitchen and we termed it as chef cook mode; Type 2: the BBQ food was mainly cooked by customer themselves on the table and we termed it as customer self-service.

Each subject provided informed consent and completed a face-to-face questionnaire which included demographic data, lifestyle information and an occupational history. These subjects all agreed to donate blood and urine samples during their routine physical examination. Blood was collected into both K2-EDTA and heparin vacutainer tubes. Urine was collected into sterile sample cups. All samples were maintained on ice packs during transportation to the laboratory. Then the blood samples were immediately processed for each test and urine samples were aliquoted and stored at − 80 °C until analysis.

### RBC *PIG-A* assay

The samples were analyzed by a flow cytometer equipped with a 488-nm laser (Accuri C6, v1.0.264.21 software, BD, San Jose, CA). Erythrocyte staining and the flow cytometer gating strategy for *PIG-A* MFs and % RET analysis were described previously [8]. In short, 3 μL of K2-EDTA anticoagulated whole blood were stained with 2.5 μL of 10-fold diluted APC Mouse Anti-Human CD235a (BD Biosciences, Cat No: 551336); and then, 20 μL FITC Mouse Anti-Human CD59 p282(H19) (BD Biosciences, Cat No: 555763) were added for the *PIG-A* assay, or 20 μL PE Mouse Anti-Human CD71 (BD Biosciences, Cat No: 555537) for the % RET assay. Two million cells were analyzed per subjects for the *PIG-A* assay, and a *PIG-A* mutant frequency was calculated as the number of *PIG-A* mutant cells per million erythrocytes (10^− 6^). While, at least 2 × 10^5^ erythrocytes were evaluated for high CD71-PE expression to calculate % RET.

### Lymphocyte CBMN test

The lymphocyte CBMN test was carried out according to previously described methods and scoring criteria [30–32]. Briefly, we added 0.5 mL heparinized blood into 4.5 mL of RPMI-1640 medium with phytohemagglutinin (PHA) and 10% fetal bovine serum. The cell culture was incubated at 37 °C with 5% CO2 for 44 h and then treated with 6 μg/mL Cytochalasin B to block cytokinesis for an additional 28 h. At the end of the incubation, the cells were harvested by centrifugation and treated with 0.075 M KCl. Then the cells treated with fixative (fresh mixed methanol: acetic acid 3:1 v/v). The lymphocytes in fresh fixative were dropped onto clean slides, air-dried and stained with 10% Giemsa (pH 6.8) for 10 min. Two thousand binucleated (BN) lymphocytes were analyzed for each subject to determine the frequencies of micronuclei (MN), nucleoplasmic bridges (NPB) and nuclear buds (NBUD).

### Determination of urinary PAHs metabolite

Urinary hydroxylated PAHs (OH-PAHs), the internal biomarker of PAHs, including 1-hydroxy-naphthalene (1-OH-NaP), 2-hydroxy-naphthalene (2-OH-NaP), 3-hydroxy-fluorene (3-OH-Flu), 2-hydroxy-fluorene (2-OH-Flu), 3-hydroxy-phenanthrene (3-OH-Phe), 1-hydroxy-phenanthrene (1-OH-Phe), 2-hydroxy-phenanthrene (2-OH-Phe), 9-hydroxy-phenanthrene (9-OH-Phe), 4-hydroxy-phenanthrene (4-OH-Phe), and 1-hydroxy-pyrene (1-OH-Pyr), were determined by on-line solid-phase extraction-high performance liquid chromatography-tandem mass spectrometry (on-line SPE-HPLC-MS/MS. Briefly, after isotope-labeled internal standards (d_7_–2-OH-NaP, d_9_–2-OH-Flu, d_9_–1-OH-Pyr, and ^13^C-3-OH-Phe) added, 500 μL urine sample was incubated with β-glucuronidase (Sigma-Aldrich) in the dark at 37 °C over night. After adding 500 μL acetonitrile (Merck, Darmstadt, Germany) to precipitate protein, the mixture was centrifuged with 12,000 rpm for 10 min at 4 °C and the supernatant was transfer for further analysis. Sample extracts was performed using a two position six-way valve (G1170A 1290 valve drive, Agilent, Santa Clara, CA, USA) and detected by the triple quadrupole mass spectrometer (Agilent, Santa Clara, CA, USA).

### Statistical analysis

We performed statistical analyses using the SAS software package version 9.4 (SAS Institute, Cary, NC). Two-sided *P*-values less than 0.05 were considered statistically significant. For statistical evaluations, *PIG-A* MF frequencies were log (10) transformed, and a square root transformation was applied to the frequencies of MN, NBP and NBUD. Multivariate Poisson regression analysis was used to compare the *PIG-A* MFs between the PAH-exposed workers and the controls, or the frequency of MN, NPB and NBUD, and for estimating the relative risk (RR) and its 95% confidence intervals (CIs). The Spearman test was performed to assess correlations between urinary PAHs metabolites and *PIG-A* MF and the frequencies of MN, NPB and NBUD. The Kruskal-Wallis test was used to compare *PIG-A* MF among the controls, the low and high PAHs-exposed groups. A Chi-square test for trend was performed to assess the association between *PIG-A* MF and PAHs exposure level. The Mann-Whitney non-parametric test was used to compare the differences compare the differences in *PIG-A* MF for smokers and non-smokers; drinkers and non-drinkers; the workers in Type 1 and 2 restaurant; respectively. All the graphs were potted using GraphPad Prism (Prism Software, version 8.02, Nashville, TN).

## Results

We recruited 70 BBQ restaurant workers (i.e., the PAH-exposed group), including 39 men and 31 women (mean age, 31.5 ± 11.9, range: 18–55 years), and 56 hotel administrative staff (i.e., the non-exposed control group), including 25 men and 31 women (mean age, 30.1 ± 9.7, range: 18–54 years). The demographics and characteristics of the subjects are listed in Table 1.

**Table 1:** Characteristics of the occupational PAHs-exposed workers (*N* = 70) and hotel administrative staffs(*N* = 56)

		Number (%)	
		Controls(***N*** = 56)	PAHs-exposed workers(***N*** = 70)
**Sex**	Male	25 (44.6)	39 (55.7)
	Female	31 (55.4)	31 (44.3)
**Age (years)**	18 ≤ age < 30	33 (58.9)	38 (54.3)
	30 ≤ age < 40	12 (21.4)	11 (15.7)
	40 ≤ age < 50	9 (16.1)	12 (17.1)
	50 ≤ age ≤ 60	2 (3.6)	9 (12.9)
**Years of work** *Median (P25,P75)*		–	0.5 (1.0, 2.0)
**Smoking status**	Yes	11 (19.6)	20(28.6)
	No	45 (80.4)	50 (71.4)
**Alcohol use**	Yes	9 (16.1)	10(14.3)
	No	47(83.9)	60 (85.7)

**Fig. 1:**
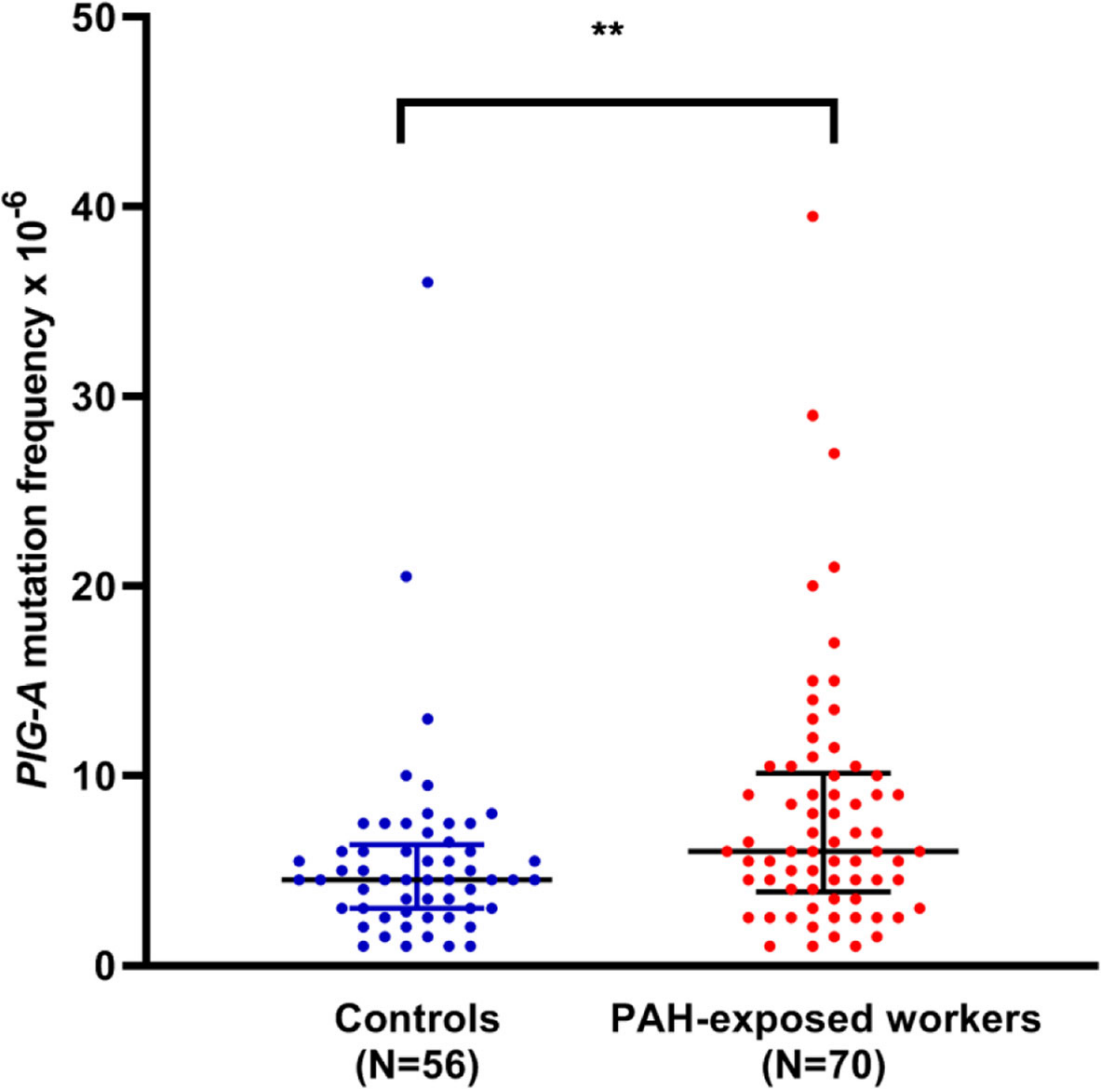
The comparison of *PIG-A* MFs between PAH-exposed workers (*N* = 70) and controls (*N* = 56). Black line: median with interquartile range. The average *PIG-A* MF for the PAH-exposed workers was 8.04 ± 6.81 × 10^− 6^ (median: 6.00 × 10^− 6^; interquartile range: 3.88–10.13 × 10^− 6^; range: 1.00–39.50 × 10^− 6^). The *PIG-A* MF for the control group was 5.56 ± 5.26 × 10^− 6^ (median: 4.50 × 10^− 6^; interquartile range: 3.00–6.38 × 10^− 6^; range: 1.00–36.00 × 10^− 6^). **Significant differences *P* < .01

As shown in Fig. 1**,** the average *PIG-A* MF for the PAH-exposed workers was 8.04 ± 6.81 × 10^− 6^ (median: 6.00 × 10^− 6^; interquartile range: 3.88–10.13 × 10^− 6^; range: 1.00–39.50 × 10^− 6^). The *PIG-A* MF for the control group was 5.56 ± 5.26 × 10^− 6^ (median: 4.50 × 10^− 6^; interquartile range: 3.00–6.38 × 10^− 6^; range: 1.00–36.00 × 10^− 6^). Multivariate Poisson regression analysis (adjusted for age and sex) showed that the PAH-exposed workers had a significantly higher *PIG-A* MF than the control subjects (RR = 0.707, 95% confidence interval [CI]: 0.615–0.812, *P* < 0.001). The % RET (based on the % RET assay) for the PAH-exposed workers and the control subjects ranged from 0.02–0.62% and 0.01–0.90%, respectively, suggesting abnormalities were not present in subjects.

The average *PIG-A* MF for the male PAH-exposed workers (8.56 ± 6.12 × 10^− 6^) was slightly higher than that of the female PAH-exposed workers (7.39 ± 7.64 × 10^− 6^); however, this difference was not significant (*P* = 0.093). The average *PIG-A* MF for the male control subjects (6.12 ± 6.88 × 10^− 6^) was also slightly higher than that of the female control subjects (5.11 ± 3.51 × 10^− 6^); however, this difference was not significant (*P* = 0.886).

Spearman analysis showed that *PIG-A* MFs were not associated with age in the PAH-exposed workers (*P* = 0.969, *r* = 0.005) or the control subjects (*P* = 0.345, *r* = 0.129), and also showed no association with years of work (*P* = 0.116, *r* = 0.200) in the PAH-exposed workers. In addition, Mann-Whitney analysis conducted on the PAH-exposed workers indicated that *PIG-A* MFs did not differ between smokers (6.50 ± 3.5 × 10^− 6^, *N* = 20) and non-smokers (8.66 ± 7.7 × 10^− 6^, *N* = 50) (*P* = 0.701), or between drinkers (6.55 ± 4.2 × 10^− 6^, *N* = 10) and non-drinkers (8.29 ± 7.1 × 10^− 6^, *N* = 60) (*P* = 0.674). In control subjects, *PIG-A* MFs also showed no difference between smokers (8.68 ± 7.6 × 10^− 6^, *N* = 11) and non-smokers (4.80 ± 3,2 × 10^− 6^, *N* = 45) (*P* = 0.116), or between drinkers (5.94 ± 2.7 × 10^− 6^, *N* = 9) and non-drinkers (5.49 ± 5.6 × 10^− 6^, *N* = 47) (*P* = 0.140).

### The PAH-exposed workers exhibited significantly elevated MN and NBUD frequencies compared with the control subjects

**Fig. 2:**
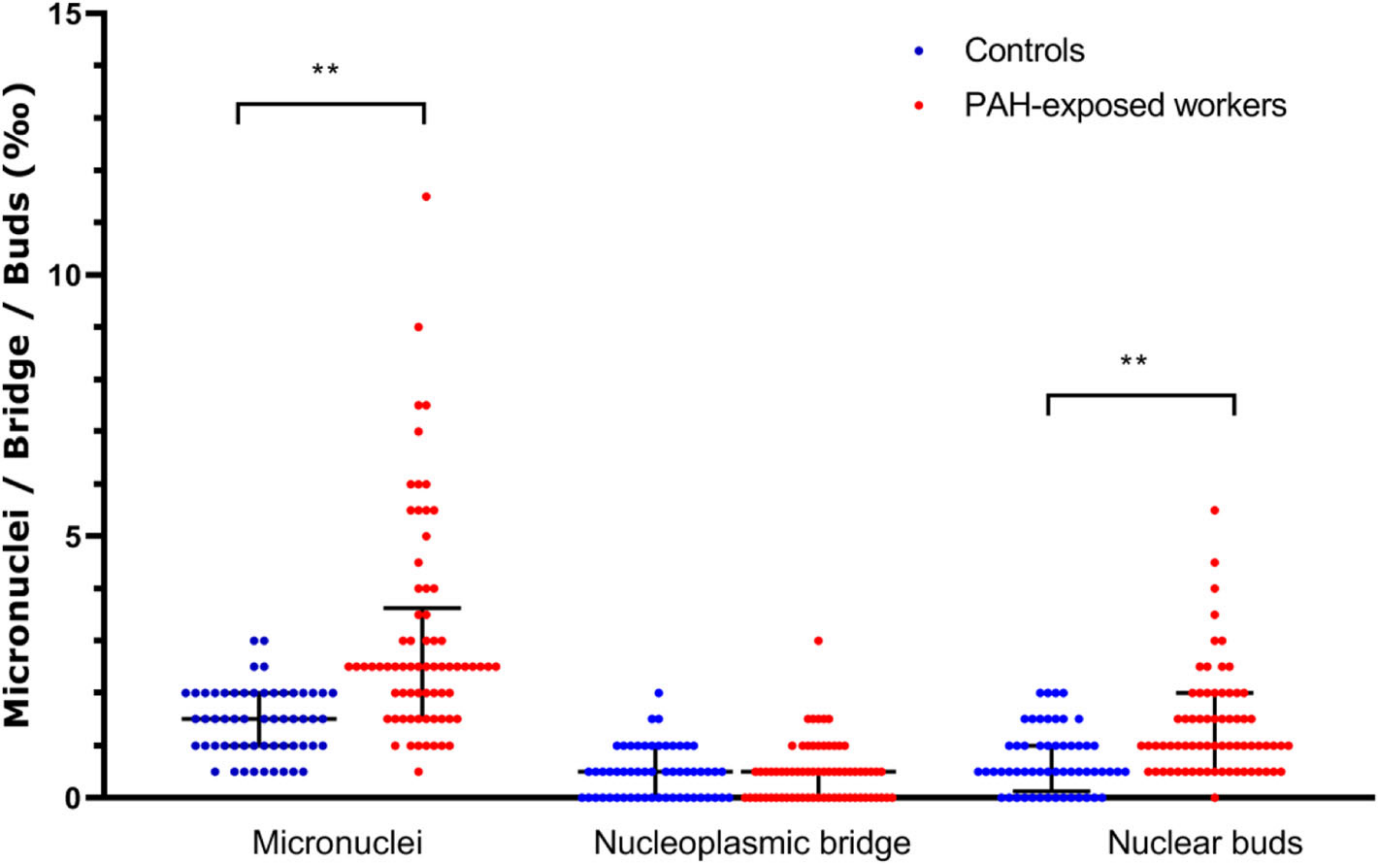
Frequencies of Micronuclei (MN), nucleoplasmic bridges (NPB) and nuclear buds (NBUD) in PAH-exposed workers (*N* = 70) and controls (*N* = 56). Black line: median with interquartile range. Among the PAH-exposed workers, MN, NPB, and NBUD frequencies were 3.06 ± 2.07 ‰, 0.47 ± 0.55 ‰, and 1.38 ± 1.02 ‰, respectively. Among the control subjects, MN, NPB, and NBUD frequencies were 1.46 ± 0.64 ‰, 0.47 ± 0.48 ‰, and 0.70 ± 0.60 ‰, respectively. **Significant differences *P* < .01

As shown in Fig. 2**,** among the PAH-exposed workers, MN, NPB, and NBUD frequencies were 3.06 ± 2.07 ‰, 0.47 ± 0.55 ‰, and 1.38 ± 1.02 ‰, respectively. Among the control subjects, MN, NPB, and NBUD frequencies were 1.46 ± 0.64 ‰, 0.47 ± 0.48 ‰, and 0.70 ± 0.60 ‰, respectively. Multivariate Poisson regression analysis (adjusted for age, sex, and smoking status) showed that the PAH-exposed workers had significantly higher MN and NBUD frequencies than the control subjects (MN frequency: RR = 0.497, 95% CI: 0.383–0.641, *P* < 0.001; NBUD frequency: RR = 0.548, 95% CI: 0.421–0.713, *P* < 0.001).

Among the PAH-exposed workers, the average MN, NPB, and NBUD frequencies in male subjects were 2.55 ± 1.79 ‰, 0.38 ± 0.45 ‰, and 1.10 ± 0.71 ‰, respectively, and the average MN, NPB, and NBUD frequencies in female subjects were 3.71 ± 2.24 ‰, 0.58 ± 0.65 ‰ and 1.73 ± 1.23 ‰, respectively. The MN and NBUD frequencies for females were slightly higher than those for males (MN frequency: *P* = 0.013; NBUD frequency: *P* = 0.015).

Among the control subjects, the average MN, NPB, and NBUD frequencies in male subjects were 1.34 ± 0.64 ‰, 0.38 ± 0.51 ‰, and 0.92 ± 0.62 ‰, respectively, and the average MN, NPB, and NBUD frequencies in female subjects were 1.53 ± 0.64 ‰, 0.55 ± 0.45 ‰ and 0.52 ± 0.53 ‰, respectively. The NBUD frequencies for males were slightly higher than those for females (*P* = 0.017).

### *PIG-A* MF had a better association with OH-PAH metabolites than MN, NPB and NBUD frequencies did in urine

**Fig. 3:**
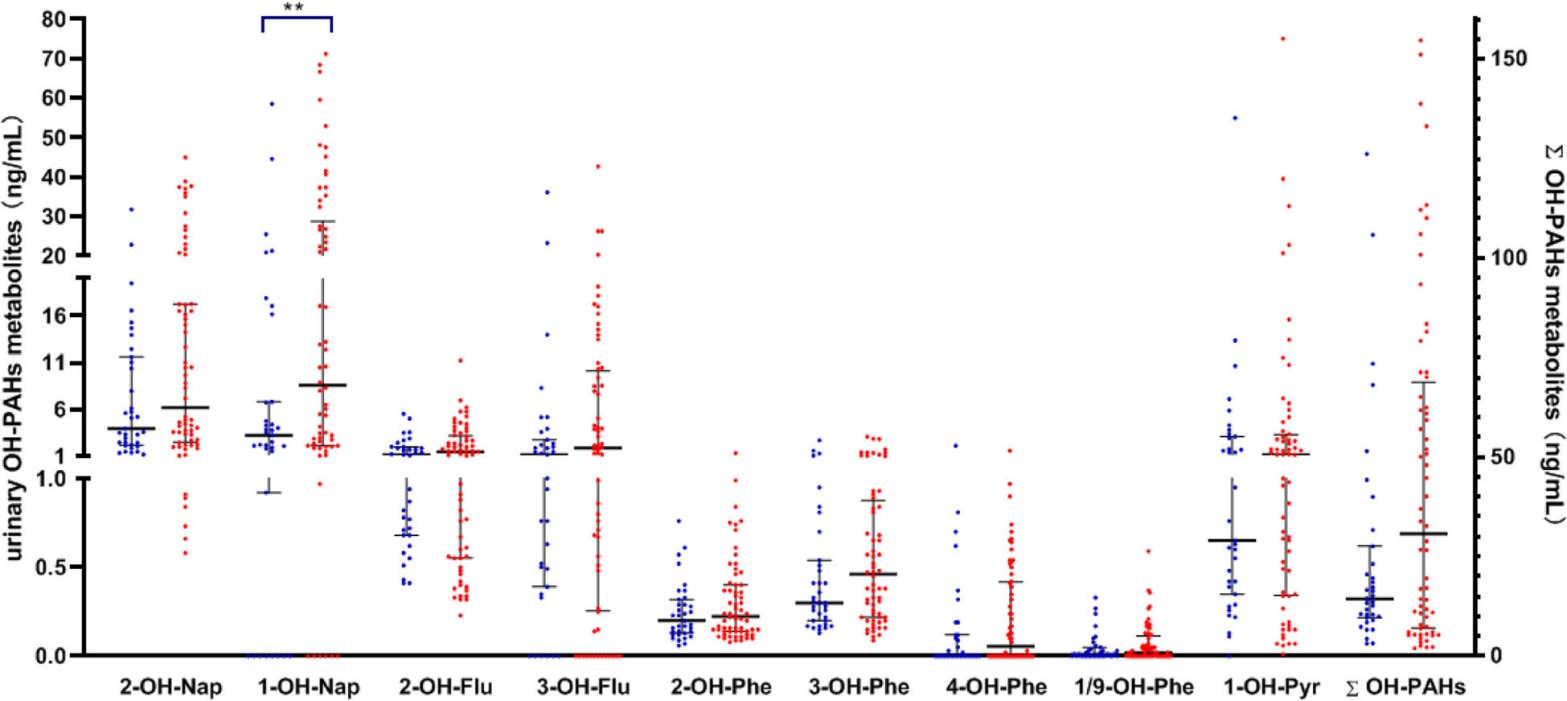
Concentrations (ng/mL) of urinary OH-PAHs metabolites in PAH-exposed workers (*N* = 64) and the control group (*N* = 35). Blue dot the control group, red dot: PAH-exposed group, black line: median with interquartile range. Mann-Whitney analysis showed that only the 1-OH-Nap concentration was significantly higher (*P* = 0.009). **Significant differences *P* < .01

Urinary OH-PAH metabolites were detected in 64 PAH-exposed workers and 35 control subjects due to limitations (Fig. 3 and Table S1). Although the mean and median values of all the OH-PAH metabolites were higher in the PAH-exposed workers than in the control subjects, Mann-Whitney analysis showed that only the 1-OH-Nap concentration was significantly higher (*P* = 0.009).

The increase in *PIG-A* MF in the PAH-exposed workers was associated with OH-PAH metabolites of 2-OH-Flu (*P* = 0.032, *r* = 0.268), 2-OH-Phe (*P* = 0.022, *r* = 0.286), 3-OH-Phe (*P* = 0.0312, *r* = 0.270), and 4-OH-Phe (*P* = 0.018, *r* = 0.296). However, the increase in MN, NPB, and NBUD frequencies was not associated with any OH-PAH metabolites.

Furthermore, compared with the controls and the low PAH-exposed group, the Kruskal-Wallis test showed that the high PAH-exposed group had a statistically significant highest concentrations of all urinary OH-PAH metabolites (*P*_2-OH-Nap_ = 0.018, *P*_1-OH-Nap_ < 0.001, *P*_2-OH-Flu_ = 0.005, *P*_3-OH-Flu_ < 0.001, *P*_2-OH-Phe_ = 0.007, *P*_*3*-OH-Phe_ = 0.013, *P*_4-OH-Phe_ = 0.011, *P*_1/9-OH-Phe_ = 0.025, *P*_1-OH-Pyr_ = 0.008, *P*_ΣOH-PAHs_ = 0.002).

### High-PAH-exposed workers showed the highest *PIG-A* MFs

For further analysis the relationships between the levels of PAH exposure and *PIG-A* MF, we divided the 70 BBQ workers into the high PAH-exposed group (all chefs) and low PAH-exposed group (other types of work) according to the different types of work. The *PIG-A* MF for the high PAH-exposed group ((*N* = 24) was 9.85 ± 6.98 × 10^− 6^ (median: 8.50 × 10^− 6^; interquartile range: 5.50–11.63 × 10^− 6^; range: 1.50–29.00 × 10^− 6^), and the *PIG-A* MF for low PAH-exposed group (*N* = 46) was 7.10 ± 6.60 × 10^− 6^ (median: 5.25 × 10^− 6^; interquartile range: 2.50–9.25 × 10^− 6^; range: 1.00–39.50 × 10^− 6^). Multivariate Poisson regression analysis (adjusting for age, sex, and smoking status) showed that the high PAH-exposed group had a significantly higher *PIG-A* MF than the low PAH-exposed group (RR = 0.738, 95% CI: 0.637–0.854, *P* < 0.001).

We used a *PIG-A* MF value of 9.25 × 10^− 6^ as the upper limit for a normal *PIG-A* MF [8] and considered *PIG-A* MFs greater than 9.25 × 10^− 6^ as abnormal to further analyze the association between *PIG-A* MF and PAH exposure. Using this threshold, we identified 51, 35, and 16 subjects with a normal *PIG-A* MF in control, low PAH-exposed, and high PAH-exposed groups, respectively. The Chi-square test using these data indicated a significant association between *PIG-A* MF and PAH exposure levels (*P* = 0.006).

### The PAH-exposed workers in a customer self-service BBQ restaurant showed higher *PIG-A* MFs

We compared the *PIG-A* MFs between the subjects working in Type 1 (chef cook mode) and Type 2 (customer self-service) restaurants because we considered that the ambient PAH levels in the two types of restaurants were different. Mann-Whitney analysis showed that the subjects working in Type 2 BBQ restaurants had a higher *PIG-A* MF (10.32 ± 8.33 × 10^− 6^, median: 8.00 × 10^− 6^, *N* = 28) than those in Type 1 BBQ restaurants (6.52 ± 5.13 × 10^− 6^, median: 5.00 × 10^− 6^, *N* = 40) (*P* = 0.014). However, the results of the CBMN test did not show any differences. Mann-Whitney analysis showed that only the 1/9-OH-Phe concentration was significantly higher in subjects working in the Type 2 BBQ restaurant (*P* = 0.046).

## Discussion

In the present study, the human *PIG-A* assay determined increases in *PIG-A* mutant levels in the PAH-exposed subjects. We found that *PIG-A* MFs in 70 PAH-exposed workers were significantly higher than those in the 56 control subjects. The estimated RR was 0.707, showing that the control subjects had a lower risk of inducing *PIG-A* mutations. Additionally, the high-PAH-exposed workers exhibited the highest *PIG-A* MFs, and the Chi-square test for trend also indicated a significant association between *PIG-A* MF and PAH exposure levels.

The *PIG-A* MFs of the control subjects in our study were consistent with our published data [8, 13] and others [6, 7]. Meanwhile, there were no correlations between age and *PIG-A* MF for the PAH-exposed workers or the control subjects, consistent with previous studies [6, 8]. Other studies have shown conflicting results [7, 9], and a study by Lawrence et al., with a large data set (300 subjects), found positive correlations with age and *PIG-A* MF. They speculated that individuals accumulate mutations over time and have reduced DNA repair capacity [9]. The subjects in our studies (including our previously published studies) were mostly young and middle-aged, which might not be the most suitable model to explore the correlation between age and *PIG-A* MF. In addition, there were no statistically significant differences in *PIG-A* MF between smokers and non-smokers or between drinkers and non-drinkers. Dobrolsky et al. and Lawrence et al. did not find such a difference in smoking status [6, 9], but another study by Haboubi et al. showed conflicting results [10]. These discrepancies could be due to the disproportionate number of smokers and non-smokers in these studies, and the smoking status might be easily influenced by other confounding factors.

In addition, *PIG-A* MF had a better association with OH-PAH metabolites than MN, NPB and NBUD frequencies did in urine. However, we acknowledge that the correlation coefficient r values in Spearman analysis were around 0.3, exhibiting a weak positive relationship between *PIG-A* MF and the corresponding OH-PAH metabolites. Our results showed that the *PIG-A* assay might better reflect the response of PAH exposure, possibly due to *PIG-A* MFs reflecting the accumulation of mutagenic damage, consistent with our previous work [33]. Nevertheless, we also found that the PAH-exposed workers exhibited significantly elevated MN and NBUD frequencies compared with the control subjects, similar to those published previously [22, 34]. The MN frequencies of the PAH-exposed workers (3.06 ± 2.07 ‰) did not seem to be particularly high, but the data are similar to those of studies with similar internal exposure data of urinary 1-OH-Pyr levels [35–37]. The MN frequencies of lymphocytes in the CBMN test reflect the extent of DNA damage remaining at the time of detection because in vivo DNA damage is usually repairable. These lymphocytes are mostly T-cells, including long-lived “memory” cells and short-lived uncommitted naïve cells (half-life is a few days), and MN frequencies in lymphocytes may provide lesion measures over a short-term period, considering the lifespan and self-renewal ability of the circulating lymphocytes. Whereas the RBC lineage derives from early-stage hematopoietic progenitor cells or hematopoietic stem cells in the bone marrow, the RBC *PIG-A* assay quantitatively reflects the consequences of earlier mutations in hematopoietic progenitor cells or hematopoietic stem cells, determining the accumulated long-term exposure. On the other aspect, the MN test mainly detects aneugenicity/clastogenicity, whereas the * PIG-A* assay detects gene mutations, and the combination of different genetic endpoints will better inform health risk assessments.

In our study, we measured ten representative urinary hydroxylated metabolites, including naphthalene, fluorine, phenanthrene, and pyrene, to evaluate occupational PAH exposure [38]. In the present study, only 1-OH-Nap exhibited significantly higher levels in the PAH-exposed workers than in the control subjects, possibly attributed to the relatively low PAH metabolites urine levels in workers during the BBQ occupational exposure [39]. Moreover, urinary PAH metabolites may reflect acute exposure to PAHs since the half-life for urinary excretion of 1-OH-Pyr, the most commonly used biomarker [40], is approximately 18 h and ranges from 6 to 35 h [41, 42]. Besides that, the ∑OH-PAH of the high PAH-exposed group (median: 62.24 ng/mL, interquartile range: 15.69–107.76 ng/mL) was about four folds higher than the control group (median: 14.48 ng/mL, interquartile range: 9.71–27.5 ng/mL). According the reference [43], the median of urinary concentrations of ∑OH-PAH among the general population in urban of Guangdong, south China was 12.7 (interquartile range: 4.98–31.7 ng/mL) which was consist with the data of control group in our study. We considered that the high PAH-exposed group (all chefs) showed a biological significance higher concentrations of all urinary OH-PAH metabolites compared with the control subjects and the low PAH-exposed group (other types of work).

An interesting finding in this study was that *PIG-A* MFs showed differences between workers from different cooking restaurants. For instance, *PIG-A* MFs in workers from the Type 2 BBQ restaurants (customer self-service) were higher than those in the Type 1 BBQ restaurants (chef cook mode). We speculate that it maybe because food was mainly cooked by the customers on the table In the Type 2 restaurants and most of them used charcoal to cook food, so it filled the entire interior space with smoke and facilitating PAHs exposure to all the workers. The concentration of PAHs might be very high in these small space restaurants. In contrast, food was generally cooked under ventilation systems in the kitchen in Type 1 restaurants. We speculate that the other types of workers except chefs may have been more exposed to PAHs in Type 2 restaurants than in Type 1 restaurants. In addition, the pollutant levels and compositions in BBQ restaurants can vary according to the cooking fuels, cooking mode, ventilation systems, etc., used during food preparation. Further studies are needed to investigate external exposure levels by carrying personal air samplers and stationary samplers measuring airborne contaminants (e.g., heavy metals, airborne carbonyls, fine particulate matter (PM_2.5_) [44]). In addition, a more detailed questionnaire (e.g., BBQ food intake, urinary sample collection time) and assessing the influence of work year with external exposure biomarkers will facilitate the evaluation of the toxic effects of PAHs.

## Conclusion

In conclusion, the PAH occupational exposure population study results suggest that the *PIG-A* gene mutation is a promising genotoxicity biomarker applicable to human biomonitoring studies.

## Supplementary Information

**Additional file 1.** Supporting information.

## Data Availability

The data used and analyzed in the present study are available from the corresponding author on reasonable request.
