# Supplementary material for: PIG-A gene mutation as a genotoxicity biomaker in polycyclic aromatic hydrocarbon-exposed barbecue workers
Source: Genes Environ. 2021 Dec 9;43:54. doi: 10.1186/s41021-021-00230-1 (PMC8656086; doi:10.1186/s41021-021-00230-1)
Supplement: Supplementary file 1 — Additional file 1. Supporting information. [file 41021_2021_230_MOESM1_ESM.docx]

**SUPPORTING INFORMATION**

**Table S1. Concentrations (ng/mL) of urinary OH-PAHs metabolites in PAHs-exposed workers (N =64) and the control group (N=35）**

|  | **2-OH-Nap** | **1-OH-Nap** | **2-OH-Flu** | **3-OH-Flu** | **2-OH-Phe** | **3-OH-Phe** | **4-OH-Phe** | **1/9-OH-Phe** | **1-OH-Pyr** | **ΣOH-PAHs** |
| --- | --- | --- | --- | --- | --- | --- | --- | --- | --- | --- |
| **PAHs-exposed workers （N=64; the number of -OH-Nap is 62）** | | | | | | | | | | |
| **Mean** | 12.1 | 17.75 | 2.114 | 6.03 | 0.3117 | 0.6506 | 0.2255 | 0.07 | 5.056 | 43.76 |
| **Std. Deviation** | 12.13 | 19.79 | 2.103 | 8.452 | 0.2461 | 0.637 | 0.3173 | 0.1116 | 11.64 | 42.06 |
| **Median** | 6.195 | 8.58 | 1.45 | 1.86 | 0.225 | 0.46 | 0.055 | 0.02 | 1.125 | 30.75 |
| **Minimum** | 0.58 | <LOD | 0.23 | <LOD | 0.08 | 0.09 | <LOD | <LOD | 0.01 | 1.95 |
| **25% Percentile** | 2.503 | 2.12 | 0.5525 | 0.255 | 0.14 | 0.22 | <LOD | <LOD | 0.3425 | 7.095 |
| **75% Percentile** | 17.26 | 28.8 | 3.18 | 10.09 | 0.4 | 0.8775 | 0.4175 | 0.115 | 3.283 | 68.87 |
| **Maximum** | 45.06 | 71.26 | 11.25 | 42.77 | 1.3 | 3.07 | 1.58 | 0.59 | 75.1 | 154.7 |
| **Detection frequency(%)** | 100.0 | 90.3 | 100.0 | 79.7 | 100.0 | 100.0 | 53.1 | 57.8 | 100.0 |  |
| **Control group (N=35)** | | | | | | | | | | |
| Mean | 7.398 | 8.132 | 1.566 | 3.562 | 0.248 | 0.4991 | 0.1669 | 0.04629 | 3.59 | 25.21 |
| Std. Deviation | 7.219 | 13.04 | 1.259 | 7.268 | 0.1638 | 0.5137 | 0.3974 | 0.08286 | 9.436 | 28.4 |
| Median | 3.95 | 3.24 | 1.14 | 1.15 | 0.2 | 0.3 | <LOD | 0.01 | 0.65 | 14.48 |
| Minimum | 1.17 | <LOD | 0.41 | <LOD | 0.06 | 0.13 | <LOD | <LOD | <LOD | 3.14 |
| 25% Percentile | 2.2 | 0.92 | 0.68 | 0.39 | 0.13 | 0.2 | <LOD | <LOD | 0.35 | 9.71 |
| 75% Percentile | 11.63 | 6.81 | 1.97 | 2.76 | 0.32 | 0.54 | 0.12 | 0.05 | 3.09 | 27.59 |
| Maximum | 31.81 | 58.53 | 5.53 | 36.17 | 0.76 | 2.7 | 2.11 | 0.33 | 54.97 | 126.2 |
| Detection frequency(%) | 100.0 | 77.1 | 100.0 | 82.9 | 100.0 | 100.0 | 42.9 | 54.3 | 97.1 |  |
